# The Combined Use of Inflammation Markers, Modified Glasgow Prognostic Score, and Sarculator Nomogram in Extremity Soft Tissue Sarcoma: A Multicenter Observational Study

**DOI:** 10.3390/cancers16051077

**Published:** 2024-03-06

**Authors:** Tomoki Nakamura, Satoshi Takenaka, Hidetatsu Outani, Tomohito Hagi, Hironari Tamiya, Yoshinori Imura, Kunihiro Asanuma, Akihiro Sudo

**Affiliations:** 1 Department of Orthopaedic Surgery, Mie University Graduate School of Medicine, Tsu 514-8507, Japan; hagifana@med.mie-u.ac.jp (T.H.); k-asanum@clin.medic.mie-u.ac.jp (K.A.); a-sudou@med.mie-u.ac.jp (A.S.); 2 Musculoskeletal Oncology Service, Osaka International Cancer Institute, Osaka 541-8567, Japan; s.takenaka.0816@gmail.com (S.T.); tamiyahironari@yahoo.co.jp (H.T.); 3 Department of Orthopedic Surgery, Osaka University Graduate School of Medicine, Suita 565-0871, Japan; h-otani@ort.med.osaka-u.ac.jp (H.O.); great_neo_universe@yahoo.co.jp (Y.I.)

**Keywords:** Sarculator, modified Glasgow prognostic score, soft tissue sarcoma

## Abstract

**Simple Summary:**

A total of 217 Japanese patients who underwent surgical resection for extremity STS were included. The Sarculator-predicted 10-year probability of overall survival (pr-OS) was stratified into two subgroups: lower risk (10-year pr-OS ≥ 60%) and higher risk (10-year pr-OS < 60%). The modified Glasgow prognostic score (mGPS) varied from 0 to 2. We showed that Sarculator is a validated nomogram designed to predict overall survival. Among the patients with a higher risk, those with an mGPS of 1 or 2 had poorer OS compared to those with a score of 0. The mGPS could potentially play an important role in identifying patients who are at high risk of death and metastasis in the higher-risk group on the Sarculator.

**Abstract:**

Background: Sarculator is a validated nomogram designed to predict overall survival (OS) in extremity soft tissue sarcoma (STS). Inflammation plays a critical role in cancer development and progression. There were no reports which investigated the relationship between Sarculator and inflammation. Methods: A total of 217 patients with extremity STS were included. The Sarculator-predicted 10-year probability of OS (pr-OS) was stratified into two subgroups: lower risk (10-year pr-OS ≥ 60%) and higher risk (10-year pr-OS < 60%). The modified Glasgow prognostic score (mGPS) varied from 0 to 2. Results: Out of the 217 patients, 67 were classified as higher risk, while 150 were lower risk. A total of 181 patients had an mGPS of 0, and 36 had a score of 1 or 2. The 5-year OS was 83.3%. When patients were divided into two groups according to the 10-year pr-OS, those with a higher risk had poorer OS than those with a lower risk. Among the patients with a higher risk, those with an mGPS of 1 or 2 had poorer OS compared to those with a score of 0. Conclusions: The mGPS could potentially play an important role in identifying patients who are at high risk of death and metastasis in the higher-risk group on the Sarculator.

## 1. Introduction

Soft tissue sarcoma (STS) is a rare heterogeneous tumor [1,2]. Its occurrence is relatively rare, with less than 6 per 100,000 cases, accounting for 1–2% of all adult cancer cases [1]. Patients with STS are at high risk of death and metastasis, particularly when the tumor histological grade is high, the tumor is large, and it is located deep [3]. Notably, 20–50% of patients with STS present with clinically detectable metastases, and their prognosis is commonly poor [4]. There were some nomograms for predicting survival in STS [5,6,7]. The Sarculator, especially, is a validated nomogram designed to predict overall survival (OS) in patients with extremity STS [5]. Its algorithm consists of four variables according to statistical analysis, including age, tumor size, tumor histological grade, and histological diagnosis, to predict 5- and 10-year OS and metastasis-free survival (MFS), although Japanese patients were not included in this analysis. The Sarculator is available as a free application for download. On the other hand, inflammation is deeply related to the development, progression, and clinical presentation of cancer [8]. Inflammation induces malnutrition, including hypoalbuminemia, by increasing catabolism and impairing nutrient absorption; conversely, malnutrition promotes the severity of inflammation [8,9,10]. Inflammatory cytokine, interleukin-6, contributes to the production of C-reactive protein (CRP) and the development of hypoalbuminemia [8,9,10]. Hence, it is of interest that the combination of hypoalbuminemia (<3.5 g/dL) and an elevated CRP (>1.0 mg/dL) level, used to calculate the Glasgow prognostic score (GPS), the modified GPS (mGPS), and high-sensitivity mGPS, is an important indicator [11,12,13,14]. The mGPS highlights the importance of CRP; when CRP is elevated, patients with normal albumin levels are assigned a score of 1, and those with hypoalbuminemia are assigned a score of 2 [12,13]. Recently, mGPS was reported to be a useful prognostic tool for predicting survival in 493 patients with STS in an international multicenter study [12]. In this multicenter study, the patients with an mGPS of 1 or 2 and histological high-grade sarcoma had poor survival [12]. Also, the patients with higher age and mGPS of 1 or 2 had poor survival [12]. Tumor grade and age were included in Sarculator [5]. Therefore, we hypothesized a strong relationship between the Sarculator and mGPS. Specifically, the higher-risk patients predicted by the Sarculator may have higher mGPS. There were no reports which investigated the relationship between Sarculator and inflammation. Therefore, we elucidate it in the present multicenter cooperative study.

## 2. Materials and Methods

### 2.1. Data Source

This study was approved by the Institutional Review Boards of the authors’ affiliated institutions (H2023-092). The requirement for informed consent was waived due to the nature of this study. Data from 2011 to 2020 from three hospitals were retrospectively reviewed. All patients underwent surgical resection for extremity STS. We also included patients who underwent R0 resection (no residual tumor) or R1 resection (microscopic residual tumor). Patients presenting with recurrent disease and metastases and those referred for additional resection after a previous inappropriate excision were excluded from this study. The following histologies were excluded based on their exclusion from the original Sarculator algorithm: dermatofibrosarcoma protuberans, Ewing’s sarcoma, rhabdomyosarcoma, and well-differentiated liposarcoma. The cohort included 106 men and 111 women with a mean age of 61 years (range: 20–93 years). Tumor’s location included the thigh (*n* = 120), leg (*n* = 36), upper arm (*n* = 22), forearm (*n* = 12), knee (*n* = 10), and other sites (*n* = 17). The mean follow-up period was 61 months (range 2.5–146 months). All patients underwent pretreatment staging with lung CT scans to exclude metastases. Histological diagnosis and tumor grade were determined using the French Federation of Cancer Centers Sarcoma Group grading system. Blood samples were obtained prior to treatment, including surgery, radiotherapy, and chemotherapy. R0 resection means complete tumor resection, and R1 means microscopic positive margin. The Sarculator-predicted 10-year probability of OS (pr-OS) was stratified into two subgroups: lower risk (10-year pr-OS ≥ 60%) and higher risk (10-year pr-OS < 60%). The mGPS score was calculated as previously described [10,11]. In brief, patients with both hypoalbuminemia (<3.5 g/dL) and elevated CRP levels (>1.0 mg/dL) were assigned a score of 2. Those with only elevated CRP levels were assigned a score of 1, while the rest were assigned a score of 0. Neutrophil–lymphocyte ratio (NLR) was defined as follows: the absolute neutrophil count (/μL) divided by the absolute lymphocyte count (/μL).

### 2.2. Statistical Analyses

Statistical associations between the clinicopathological variables were evaluated using the Mann–Whitney U test for quantitative data and chi-square test for qualitative data. Survival time was measured from the primary tumor’s surgery date to the date of sarcoma-related death or the last follow-up. Survival curves were generated using the Kaplan–Meier method and compared using the log-rank test. We conducted both univariate and multivariate analyses using Cox proportional hazard regression models. The variables included in the multivariate analysis were the significant factors identified in the univariate analysis. All statistical analyses were conducted using the EZR graphical user interface version 1.62 (Saitama Medical Center, Jichi Medical University, Saitama, Japan) for R (R Foundation for Statistical Computing, Vienna, Austria), a modified version of R Commander designed to add statistical functions frequently used in biostatistics.

## 3. Results

### 3.1. Patients Characteristics

The mean tumor size was 9 cm (range, 1–35 cm). The tumors were superficial in 42 patients and deep in 175 patients. Concerning STS grade, there were 28 patients with grade 1, 103 with grade 2, and 86 with grade 3 STSs, respectively. STSs were classified histologically as follows: 49 undifferentiated pleomorphic sarcomas, 32 myxoid liposarcomas (LPS), 32 myxofibrosarcomas, 27 dedifferentiated LPS, 26 leiomyosarcomas, 19 synovial sarcomas, 8 fibrosarcomas, and 24 others. All patients underwent primary surgical tumor resection. R0 resection was acquired in 188 patients, while R1 was acquired in 29 patients. NLR varied from 0.64 to 15.38. Perioperative radiotherapy and perioperative chemotherapy were received in 38 patients and 57 patients, respectively (Table 1).

The mean 10-year pr-OS rate was 66.9% (range, 3–98). Out of the 217 patients, 67 were classified as higher risk (10-year pr-OS < 60%), while 150 were classified as lower risk (10-year pr-OS < 60%). The mGPS varied from 0 to 2. A total of 181 (83.4%) patients had a score of 0, 24 (11.1%) had a score of 1, and 12 (5.5%) had a score of 2. The distribution of patients with an mGPS of 1 or 2 was larger in the high-risk group (19/68, 27.9%) than in the low-risk group (17/149, 11.4%; *p* = 0.00516, chi-squared test). The NLR was higher in patients in the higher-risk group compared to that in the lower-risk group. The median NLR in patients in the higher-risk group was 2.9, while it was 2.3 in the lower-risk group (*p* = 0.0147, Mann–Whitney U test). Furthermore, deep tumors were frequently observed in patients in the higher-risk group (62/175 = 35.4%) compared to superficial tumors (6/42 = 14.3%) (*p* = 0.00894, Mann–Whitney U test). There was no relationship between the distribution of higher risks and centers (*p* = 0.0917, chi-squared test).

### 3.2. Prognostic Factor Analyses for Overall Survival

**Figure 1:**
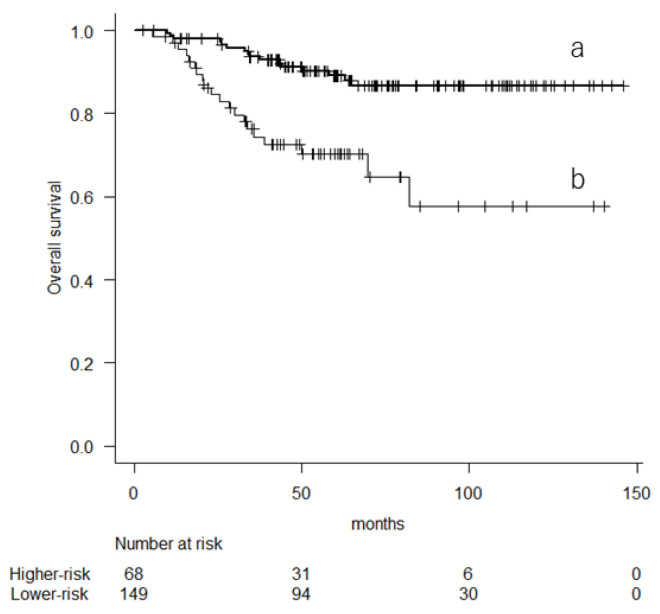
Kaplan–Meier curve showing the overall survival based on their 10-year predicted probability of overall survival (a: lower-risk group; b: higher-risk group).

At the final follow-up, 181 patients (83.4%) were alive, 29 (13.4%) had deceased due to STSs, and 7 (3.2%) had died of other causes. The 5-year OS was 83.3% (95% confidence interval (CI), 77.1–88). When patients were divided into two groups according to the 10-year pr-OS, those with a higher risk had poorer OS than those with a lower risk (*p* < 0.001, log-rank test) (Figure 1). The 5-year OS rates were 70.2% (95% CI, 56.7–80.2) for those with higher risk and 89.1% (95% CI, 82.1–93.4) for those with lower risk.

Next, when the patients were divided into two groups according to the mGPS, those with a score of 1 or 2 had poorer DSS than those with a score of 0 (*p* < 0.001, log-rank test). The 5-year OS rates were 64.9% (95% CI, 44.4–79.4) for those with a score of 1 or 2 compared with 86.7% (95% CI, 80.3–91.2) for those with a score of 0. The univariate Cox proportional hazard model showed that sex, depth, and NLR were also predictive variables for OS in addition to 10-year pr-OS and mGPS. Multivariate analysis revealed that sex and 10-year pr-OS were predictive variables for OS (Table 2).

**Figure 2:**
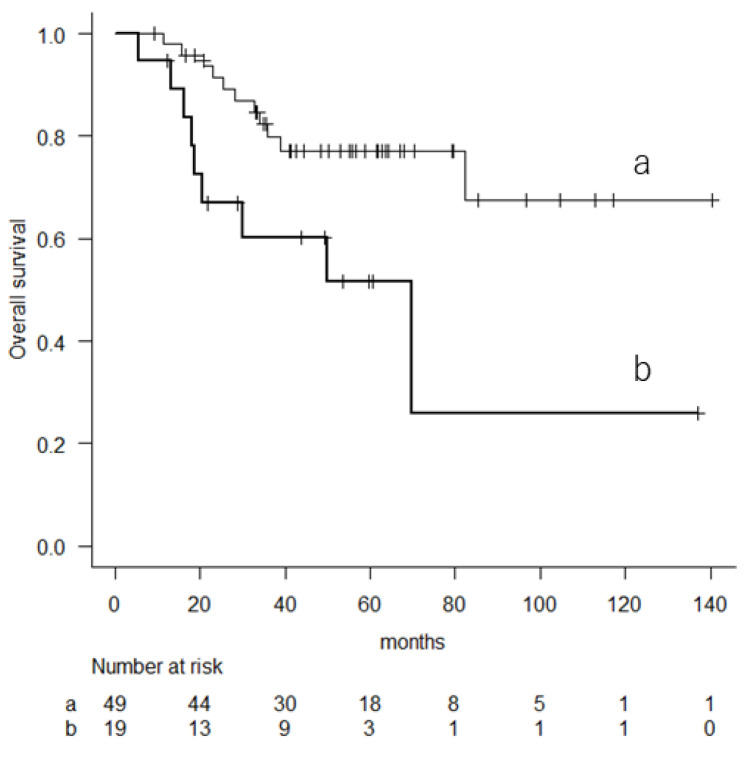
Kaplan–Meier curve showing the overall survival in patients with higher risk based on their 10-year predicted probability of overall survival (a: mGPS of 0; b: mGPS of 1 or 2).

Among the patients with a higher risk, those with an mGPS of 1 or 2 had poorer OS (5-year OS (95% CI), 51.6% (25–72.9) compared to those with a score of 0 (5-year OS (95% CI, 77.2% (61.6–87.1), *p* = 0.013, log-rank test) (Figure 2).

**Figure 3:**
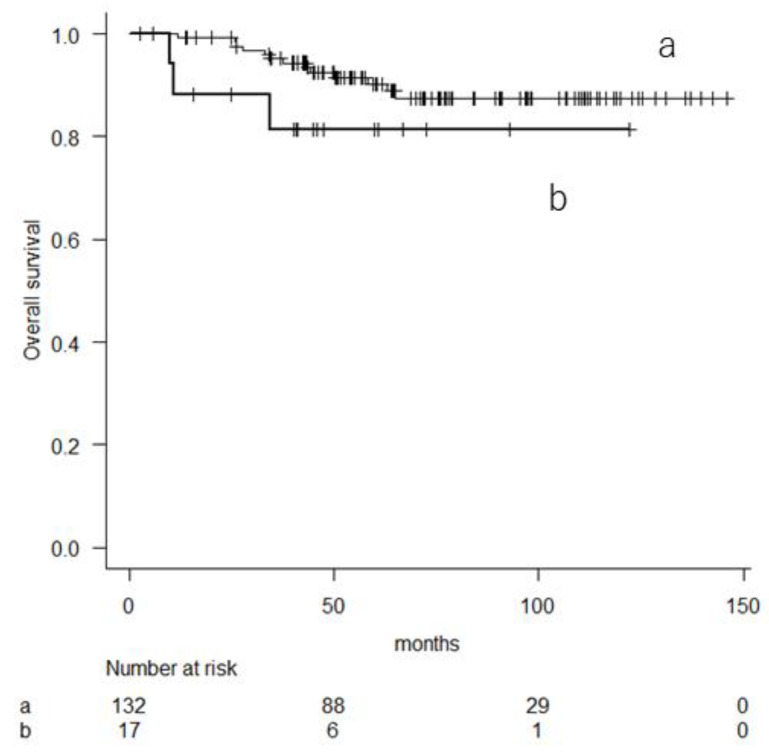
Kaplan–Meier curve showing the overall survival in patients with lower risk based on their 10-year predicted probability of overall survival (a: mGPS score of 0; b: HS-mGPS score of 1 or 2).

The multivariate Cox proportional hazard model showed that a male sex (hazard ratio (HR) 2.907, 95% CI 1.042–8.111, *p* = 0.041) and an mGPS of 1 or 2 (HR 2.49, 95% CI 1.016–6.104, *p* = 0.046) were worse prognostic variables for survival. In contrast, among patients with lower risk, there was no significant difference in OS between the group with an mGPS of 1 or 2 and a score of 0 (Figure 3). No significant variable predicted survival in this study cohort.

### 3.3. Metastatic-Free Survival and Prognostic Variables

During follow-up, 56 patients developed metastases. The 5-year MFS was 74.8% (95% CI, 68.2–80.3). Cox univariate analysis revealed that sex, 10-year pr-OS, and mGPS were prognostic variables (Table 3).

**Figure 4:**
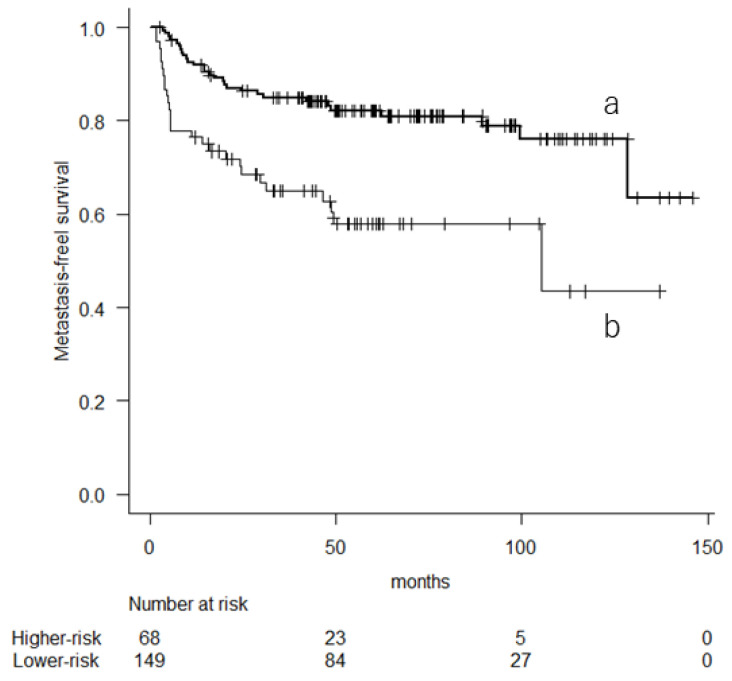
Kaplan–Meier curve showing the metastasis-free survival based on their 10-year predicted probability of overall survival (a: lower-risk group; b: higher-risk group).

The 10-year pr-OS remained significant in the multivariate analysis (*p* = 0.002). The 5-year MFS rates were 57.9% (95% CI, 44.1–69.5) for those with higher risk and 82.2% (95% CI, 74.7–87.6) for those with lower risk (Figure 4). The mGPS was a marginally significant variable (*p* = 0.06). The 5-year MFS rates were 55.3% (95% CI, 36.4–70.6) for those with an mGPS of 1 or 2 and 78.6% (95% CI, 71.5–84.2) for those with an mGPS of 0.

## 4. Discussion

The standard treatment of STS is surgical tumor resection with a wide margin [15]. This implies the removal of the tumor in a single specimen with a rim of normal tissue around it. Neo- and adjuvant radiotherapy and/or chemotherapy may be considered for patients with high-grade STS [15]. Doxorubicin and ifosfamide could be a treatment option for primary STS [16]. Patients receiving systemic chemotherapy for widely metastatic or locally advanced diseases are unsuitable for surgery or radiotherapy [17]. Doxorubicin-based chemotherapy is commonly used as first-line chemotherapy [11,12]. Pazopanib, trabectedin, and eribulin have also been administered after the failure of first-line chemotherapy [18,19,20]. However, the outcome for metastatic patients remains poor, with a median reported overall survival of 14–20 months [21,22]. Therefore, easy, well-known, and low-cost markers may help to identify a high risk of tumor relapse. Sarculator nomogram incorporated age, tumor size, grade, and histology [5]. The median 10-year pr-OS in previous studies, estimated to be 60%, was also used to generate two groups of patients to strengthen the findings of previous analyses [23,24]. Pasquali et al. revisited the EORTC-STBSG 62931 randomized controlled study and reported that patients with a predicted 10-year pr-OS lower than 60% significantly benefited from perioperative chemotherapy and surgery compared with surgery alone. Their group revisited the ISG-STS 1001 clinical trial and reported the efficacy of neoadjuvant doxorubicin and ifosfamide chemotherapy in patients with a predicted 10-year pr-OS OS lower than 60%. Therefore, we also defined patients with 10-year pr-OS lower than 60% as “higher-risk” patients in the present study. We showed that the Sarculator is a good model for predicting OS and MFS in Japanese patients with extremity STS. The multivariate analysis showed that the patients with higher risk with a 10-year pr-OS had poorer survival rates than the lower-risk group. In the present study, survival risk with a 10-year pr-OS was related to mGPS, NLR, and tumor depth. However, mGPS, NLR, and tumor depth themselves were not predictive variables for survival in the multivariate analysis. When we analyzed the predictors of survival according to higher or lower risk, we could not find any predictors of survival in patients with a lower risk. Interestingly, however, we found that mGPS may play an important role in identifying the risk of death in patients in the higher-risk group with a 10-year pr-OS. A mGPS score of 1 or 2 was frequently observed in patients with higher risk in our cohort. Voss et al. reported the utility of the Sarculator in resected extremity and trunk patients in the United States [25]. However, they found that if the Sarculator predicts a lower survival, it may be less accurate. Therefore, any indicators for enhancing higher accuracy should be necessary in patients with higher risk. No systemic inflammatory indicators were included in the analysis when the Sarculator was developed. The association between systemic inflammation and poor prognosis is well-known in patients with STSs [12,13,14]. In the tumor microenvironment, inflammation contributes to the promotion of cancer cell proliferation, invasion, and metastatic spread [26]. Several biomarkers related to inflammation, such as the NLR [27,28], GPS [12,13,14], lymphocyte-CRP ratio [29,30], systemic inflammation response index [31], and CRP-albumin ratio [32,33], have been reported to be associated with prognosis in patients with a variety of cancers, including STS. Therefore, we hypothesized that systemic inflammatory indicators may enhance the probability of survival in the Sarculator nomogram. We used mGPS because CRP and albumin are familiar to physicians, and it is easy to calculate. Our results suggest that mGPS may enhance the accuracy of predicting survival in high-risk patients. Pasquali described the efficacy of neoadjuvant chemotherapy in a higher-risk group, although we could not demonstrate superiority to survival in patients who received perioperative chemotherapy [23]. This may be due to the retrospective nature of this study and the small number of high-risk patients (16 patients) who received chemotherapy. The relationship between mGPS and the efficacy of neoadjuvant chemotherapy in high-risk patients should be confirmed in future studies.

There are some limitations in the present study. First, we only focused on the preoperative assessment of blood examinations and their derivatives. We did not evaluate postoperative changes of those because of the lack of information. Second, we performed pretreatment staging with CT scans of the lung, abdomen, and pelvis and routine blood tests to decide clinical staging and to rule out general conditions. However, not all inflammatory conditions may be detected. Relatively low patient numbers, a lack of reference histopathology, and the retrospective study design were also limitations. Therefore, prospective studies in larger patient cohorts seem warranted.

## 5. Conclusions

We confirmed that the Sarculator is an effective and valuable tool for predicting survival and metastasis in Japanese patients with extremity STS. The mGPS could play an important role in identifying patients who are at high risk of death and metastasis in the higher-risk group on the Sarculator.

## Figures and Tables

**Table 1:** Patient’s characteristics.

Variables		N
Age (years)	Mean (range)	61 (20–93)
Sex	Male	106
	Female	111
Depth	Superficial	42
	Deep	175
Tumor size (cm)	Mean (range)	9 (1–35)
Grade	1	28
	2	103
	3	86
NLR	Mean (range)	3.0 (0.64–15.38)
mGPS	0	181
	1	24
	2	12
Surgery	R0 resection	188
	R1 resection	29
Adjuvant	Chemotherapy	57
	Radiotherapy	45

NLR: neutrophil–lymphocyte ratio; mGPS: modified Glasgow prognostic score.

**Table 2:** Univariate and multivariate analysis for predicting overall survival.

		Univariate Analysis			Multivariate Analysis		
Variables		HR	95% CI	*p*-Value	HR	95% CI	*p*-Value
Sex	Female	1			1		
	Male	2.628	1.293–5.344	0.008	2.473	1.211–5.052	0.013
Depth	Deep	1			1		
	Superficial	0.22	0.053–0.918	0.037	0.335	0.078–1.432	0.14
Sarculator	Lower risk	1			1		
	Higher risk	3.533	1.823–6.845	<0.001	2.616	1.307–5.238	0.007
Albumin	≤3.5 g/dL	1					
	>3.5 g/dL	1.442	0.442–4.708	0.544			
NLR		1.151	1.009–1.314	0.036	1.041	0.894–1.213	0.604
mGPS	0	1			1		
	1 or 2	3.516	1.749–7.068	<0.001	2.109	0.968–4.595	0.06
Adjuvant Cx	No	1					
	Yes	1.095	0.528–2.273	0.808			
Adjuvant Rx	No	1					
	Yes	0.892	0.391–2.037	0.786			

NLR: neutrophil–lymphocyte ratio; mGPS: modified Glasgow prognostic score; Cx: chemotherapy; Rx: radiotherapy; HR: hazard risk; 95% CI: 95% confidence interval.

**Table 3:** Univariate and multivariate analysis for predicting metastasis.

		Univariate Analysis			Multivariate Analysis		
Variables		HR	95% CI	*p*-Value	HR	95% CI	*p*-Value
Sex	Female	1			1		
	Male	1.738	1.016–2.976	0.043	1.621	0.94–2.793	0.082
Depth	Deep	1					
	Superficial	0.8	0.391–1.636	0.541			
Sarculator	Lower risk	1			1		
	Higher risk	2.69	1.586–4.563	<0.001	2.36	1.364–4.086	0.002
Albumin	≤3.5 g/dL	1					
	>3.5 g/dL	1.542	0.614–3.874	0.357			
NLR		1.115	0.9998–1.243	0.05			
mGPS	0	1			1		
	1 or 2	2.512	1.385–4.557	0.002	1.819	0.973–3.402	0.06
Adjuvant Cx	No	1					
	Yes	1.007	0.548–1.85	0.982			
Adjuvant Rx	No	1					
	Yes	0.782	0.394–1.55	0.481			

NLR: neutrophil–lymphocyte ratio; mGPS: modified Glasgow prognostic score; Cx: chemotherapy; Rx: radiotherapy; HR: hazard risk; 95% CI: 95% confidence interval.

## Data Availability

Data are contained within the article.
